# Pancreatic cancer cell- and cancer-associated fibroblast-derived exosomes in disease progression, metastasis, and therapy

**DOI:** 10.1007/s12672-024-01111-z

**Published:** 2024-07-02

**Authors:** Yijun Chen, Jörg Kleeff, Yoshiaki Sunami

**Affiliations:** Department of Visceral, Vascular and Endocrine Surgery, Martin-Luther-University Halle-Wittenberg, University Medical Center Halle, Ernst-Grube-Str. 40, 06120 Halle (Saale), Germany

## Abstract

Exosomes play a crucial role in the progression and spread of pancreatic cancer, serving not only as promoters of tumor growth and organ-specific metastasis but also as promising biomarkers and targets for treatment. These nano vesicles enhance intercellular communication by transferring bioactive molecules, such as proteins and RNAs, between cells. This process significantly affects cancer cell dynamics, including their proliferation, migration, and invasion, while also contributing to drug resistance. Our review focuses on the crucial interactions between cancer cells and fibroblasts mediated by exosomes within the pancreatic cancer microenvironment. We delve into how exosomes from both cancer-associated fibroblasts and the cancer cells themselves drive tumor progression through various mechanisms, such as epithelial-mesenchymal transition and facilitating metastasis to specific organs like the lungs and liver. The potential of leveraging exosomes for therapeutic interventions is also explored, highlighting the importance of understanding their role in cell communication as a step forward in developing more effective pancreatic cancer treatments.

## Fundamental concepts of exosome

Exosomes are a class of small vesicles, typically ranging in diameter from 30 to 150 nm, originating from the endoplasmic reticulum, Golgi apparatus, and multivesicular bodies [1]. These nano vesicles exhibit a membrane-enclosed structure rich in various biomolecules, including proteins, lipids, and nucleic acids, making them effective mediators for intercellular communication and signal transduction [2]. Exosome formation process unfolds as follows: cellular membrane invagination initiates the creation of early endosomes. These early endosomes then progress as their membrane buds inward, evolving into late endosomes that house intraluminal vesicles. Specifically, late endosomes enriched with intraluminal vesicles are known as multivesicular bodies. Following fusion with lysosomes, some multivesicular bodies undergo degradation, while another subset fuses with the cell membrane. This fusion results in the release of intraluminal vesicles into the extracellular space, and these released vesicles are identified as exosomes [3, 4].

Initially considered as a pathway for cellular waste elimination, research has highlighted the crucial role of exosomes in intercellular communication, regulating physiological processes and disease development [5]. These small vesicles can travel through blood, bodily fluids, and other physiological fluids to distant sites from their originating cells, carrying a diverse cargo of biologically active molecules such as proteins, RNA, miRNA, and small metabolites [6]. This unique secretion mechanism has brought exosomes into the spotlight as a subject of intense research in intercellular interactions and information exchange. In-depth studies on exosomes offer robust support for understanding the molecular mechanisms of diseases and exploring novel therapeutic targets.

## Relationship between CAFs-derived exosomes and pancreatic cancer

Pancreatic cancer, characterized by its highly invasive nature, is often intricately regulated by the tumor microenvironment. Within the tumor microenvironment of pancreatic cancer, stromal cells, particularly CAFs, are recognized to play important roles in tumor development and progression. CAFs engage in complex interactions with pancreatic cancer cells e.g. through the secretion of growth factors, cytokines, chemokines, and exosomes [7]. CAF-derived exosomes can be taken up by pancreatic cancer cells, leading to changes in gene expression, thereby influencing the activation of cell signaling pathways, and regulating cellular transcription levels. This bidirectional interaction contributes to shape tumor characteristics and influence the biological behavior of pancreatic cancer.

### The relation between *cancer*-associated fibroblasts (CAF)-derived exosomes and pancreatic *cancer* cells progression

Studies have revealed that the miRNAs, proteins, and other biomarkers within exosomes derived from CAFs can influence crucial biological processes in pancreatic cancer cells [8–10]. The exosomes in the pancreatic tumor microenvironment contain RNA molecules, including but not limited to miR-10a-5p [11], miR-21 [12], miR-22 [8], miR-106b [13], miR-125b [8], miR-331-3p [14], miR-421 [15], miR616-3p [16], miR-1246 [17], miR-1290 [17], miR-3173-5p (Acyl-CoA Synthetase long chain family member 4 (ACSL4)-targeting miRNAs) [18], miR-4456 [16], miR-5703 [19], ANXA6/LRP1/TSP1 [8, 20], Hyaluronic Acid (HA) [21], tRF-19-PNR8YPJZ [22], as well as long non-coding RNA UCA1 [23] (Table 1). They promote the growth and dissemination of pancreatic cancer cells by modulating signaling pathways in the tumor microenvironment, especially those related to tumor proliferation, cell cycle control, and apoptosis. Additionally, Snail mRNA [24], a key regulator of epithelial-mesenchymal transition (EMT), along with tricarboxylic acid (TCA) cycle metabolites, amino acids, and lipids [25], also play significant roles in this process. Specific membrane proteins such as CD151 [26, 27] and Netrin-1 [28], along with the ANXA6/LRP1/TSP1 complex [8], further enhance the invasiveness and migration capabilities of pancreatic cancer cells by participating in cell–cell interactions and signaling transduction.

Exosomal miR-421, for instance, is secreted by CAFs and impacts pancreatic cancer progression by regulating a specific signaling axis. This regulatory mechanism involves the Sirtuin 3 (SIRT3), Histone H3 lysine 9 acetylation (H3K9Ac), and Hypoxia-inducible factor-1 alpha (HIF-1α) [15]. Specifically, miR-421 targets and down-regulates SIRT3, a histone deacetylase. The reduction in SIRT3 levels leads to the acetylation of H3K9, which in turn results in the up-regulation of HIF-1α. This sequence of molecular events influences the proliferation, survival, and tumorigenic potential of pancreatic cancer cells. Consequently, targeting miR-421 has emerged as a promising therapeutic strategy. Experimental studies have demonstrated that reducing the levels of miR-421 leads to a decrease in tumor growth that was initially stimulated by CAF-derived exosomes. Further emphasizing the role of CAFs in cancer progression, exosomal miR-125b-5p facilitates pancreatic cancer cell growth, migration, and invasion by suppressing the expression of the adenomatous polyposis coli (APC) gene, a known tumor suppressor [29]. This suppression leads to the activation of the Wnt signaling pathway. Moreover, a distinct subset of CAFs characterized by the expression of Netrin-G1 has been shown to produce unique exosomes that support the survival and adaptation of pancreatic cancer cells under nutritional stress [30]. The uptake of these Netrin-G1 + CAF-derived exosomes by pancreatic cancer cells activates the PI3K/Akt signaling pathway, reducing apoptosis in conditions of nutrient deficiency, thus facilitating cancer cell survival and growth under adverse conditions. Vitamin D Receptor (VDR) signaling can inhibit the release of miR-10a-5p from CAFs in their exosomes. This inhibition subsequently limits the supportive effects these CAF-derived exosomes have on the growth and development of pancreatic cancer cells [11, 31, 32]. Exosomal miR-10a-5p can be taken up by pancreatic cancer cells, and activate the TGF-β/SMAD and the Sonic Hedgehog (SHH) signaling pathways, thus enhancing the proliferation and invasion abilities of tumor cells.

These findings underscore the significant role of CAF-derived exosomal miRNAs and exosomes in the modulation of signaling pathways in pancreatic cancer cells. Understanding these interactions provides valuable insights into the mechanisms driving PDAC progression (Table 1).

**Table 1:** Exosome biomarkers in pancreatic cancer

Biomarkers	Donor	Recipiant	Exosome function	Refs
Exosomes which contribute to proliferation, migration and invasion in pancreatic cancer				
miR-10a-5p	CAFs	Tumor cells	Activate the TGF-β/SMAD and the Shh signaling pathway	[11]
miR-21	PSCs	Tumor cells	Activate the Ras/ERK pathway	[12]
miR-22, miR-125b	CAFs	Tumor cells	Reprogram cancer cells via disabling mitochondrial oxidative and increasing glycolysis	[8]
miR-106b	CAFs	Tumor cells	Block apoptosis through targeting TP53INP1 in PDAC cells	[13]
miR-125b-5p	CAFs	Tumor cells	Inhibit APC in cancer cells, activate the Wnt signaling pathway	[29]
miR-331-3p	CAFs	Tumor cells	Suppress the SCARA5/FAK axis	[14]
miR-421	CAFs	Tumor cells	Activate SIRT3/H3K9Ac/HIF-1α	[15]
miR-1246, miR-1290	Tumor cells	PSCs	Upregulation of α-SMA, production of PIP and activation of ERK, Akt signaling	[17]
miR-3173-5p (ACSL4-targeting miRNAs)	CAFs	Tumor cells	Reduce ACSL4 and inhibit ferroptosis	[18]
miR-4456, miR616-3p	PSCs	Tumor cells	Activate the PTEN/Akt pathway	[16]
miR-5703	PSCs	Tumor cells	Targeting of CMTM4, activation of PI3K/Akt pathway by PAK4	[19]
ANXA6/LRP1/TSP1	CAFs	Tumor cells	Enhanced invasiveness of pancreatic cancer	[8, 20]
Hyaluronic Acid (HA)	Tumor cells	CAFs	Elevate the secretion of MMP9 by CAFs	[21]
TCA metabolites, amino acids and lipids	CAFs	Tumor cells	Disruption of oxidative phosphorylation, increase glycolysis and glutamine-dependent reductive carboxylation	[25]
tRF-19-PNR8YPJZ	PSCs	Tumor cells	Downregulate AXIN2 and activate the Wnt signaling pathway	[22]
lncRNA UCA1	PSCs	Tumor cells	Recruitment of EZH2 and downregulation of SOCS3	[23]
Exosomes which contribute to EMT in pancreatic cancer				
ANXA6	CAFs	Tumor cells	Activate the MAPK signaling pathway	[33]
CD151	CAFs	Tumor cells	Support matrix degradation and mobility of tumor cell	[26, 27]
miR-21	PSCs	Tumor cells	Activate the Ras/ERK signaling pathway	[12]
Exosomes which contribute to chemoresistance in pancreatic cancer				
miR-146a	CAFs	Tumor cells	Resulting in pancreatic cancer cells acquiring resistance to gemcitabine and promoting cell proliferation and survival	[34]
miR-106b	CAFs	Tumor cells	Targeting TP53INP1, increase drug resistance	[8]
miR-21-5p,miR-181a-5p,miR-221-3p,miR-222-3p,miR-92a	CAFs	Tumor cells	Promote tumor cell proliferation and chemoresistance by targeting the tumor suppressor gene PTEN	[35]
miR-155	CAFs	Tumor cells	Mediating gemcitabine resistance	[36–41]
Snail mRNA	CAFs	Tumor cells	Mediating gemcitabine resistance	[24]
Snail	CAFs	Tumor cells	Activate the Akt-GSK3-Snail signaling pathway, increase drug resistance	[8, 24]

### The relation between pancreatic *cancer*-derived/CAFs-derived exosomes and EMT in pancreatic *cancer*

In the tumor microenvironment of pancreatic cancer, CAFs play a pivotal role in promoting EMT of tumor cells through the secretion of exosomes. EMT in cancer cells is a crucial biological process involved in tumor development and metastasis. During EMT, originally stationary epithelial cells acquire the ability to migrate and invade, transforming into mesenchymal-like cells. This transformation enables tumor cells to partially lose their epithelial characteristics and partially gain a mesenchymal phenotype, playing a key role in tumor initiation, progression, and metastasis [42, 43]. Additionally, EMT is associated with chemotherapy resistance, recurrence of tumors, and cancer progression [44].

Several studies aimed to identify cell type-specific as well as global exosome markers. In line with this, the proteomes of exosomes/extracellular vesicles (EVs) derived from eight distinct cell lines, encompassing PDAC cells, CAFs, and normal ductal epithelial cells, were analyzed [45]. The study detected across these cell lines various established EV markers such as alix (also known as Programed Cell Death 6-Interacting Protein), Tsg101, CD81, CD63, CD9, flotillin, integrins, and annexin V. The tetraspanins CD9, CD63 and CD81 have been widely recognized as exosome markers. CD9 is highly expressed in CAF- and pancreatic stellate cells (PSCs)-derived exosomes [46]. Another study identified putative exosome biomarkers including Syndecan-binding protein 1 (also known as synthenin-1, coded by the SDCBP gene), SLC3A2, and CD47 [46]. Notably, EVs from cancer cells and CAFs demonstrated a marked enrichment in hallmark gene sets, particularly those related to EMT, suggesting the role of EVs as mediating factors in transmitting EMT in the stromal tumor microenvironment [45]. The CAF-secreted exosomes are enriched with various bioactive molecules, such as TGF-β1 and tumor-promoting miRNAs, which can activate the TGF-β1-SMAD and WNT signaling pathways in tumor cells, thereby facilitating EMT [47, 48]. Molecules contained in CAF-derived exosomes increase the abundance of EMT markers (such as SNAIL, TWIST, and N-cadherin) in pancreatic cancer cells. These EVs secreted by CAFs, especially those carrying CD9 and ANXA6 on their surface, are taken up by pancreatic cancer cells, subsequently activating the p38 MAPK signaling pathway, leading to increased EMT of the tumor cells (Table 1) [33].

It has been shown that pancreatic cancer cells absorbing exosomes from PSCs, leading to increased miR-21 levels. This rise in miR-21 was found to enhance cell migration, trigger EMT, and boost matrix metalloproteinase-2/9 activity. Furthermore, exosomal miR-21 augmented ERK1/2 and Akt phosphorylation in these cancer cells (Table 1) [12]. These findings suggest that PSC-derived exosomal miR-21 promotes migration and EMT in pancreatic cancer cells while intensifying Ras/ERK signaling, making miR-21 a potential prognostic marker and therapeutic target for pancreatic cancer. The tetraspanins CD151 and TSPAN8 support metastatic tumor growth and metastatic niche formation in the lung and bone marrow [26, 27]. Mechanistically, exosomal CD151 and TSPAN8 are involved in integrin and matrix metalloprotease recruitment, resulting in matrix remodeling. Further, exosomes derived from CD151- and TSPAN8-competent tumor cells can be transferred into neighboring non-metastatic tumor cells, which induces expression of EMT-related genes (Table 1) [27]. In a xenograft model, rats injected with *Cd151* and *Tspan8*-knockdowned tumor cells survived significantly longer than animals with control tumor cell injection. [27]

## Pancreatic *cancer*-derived exosomes promote specific organ metastasis

### Liver metastasis

**Fig. 1:**
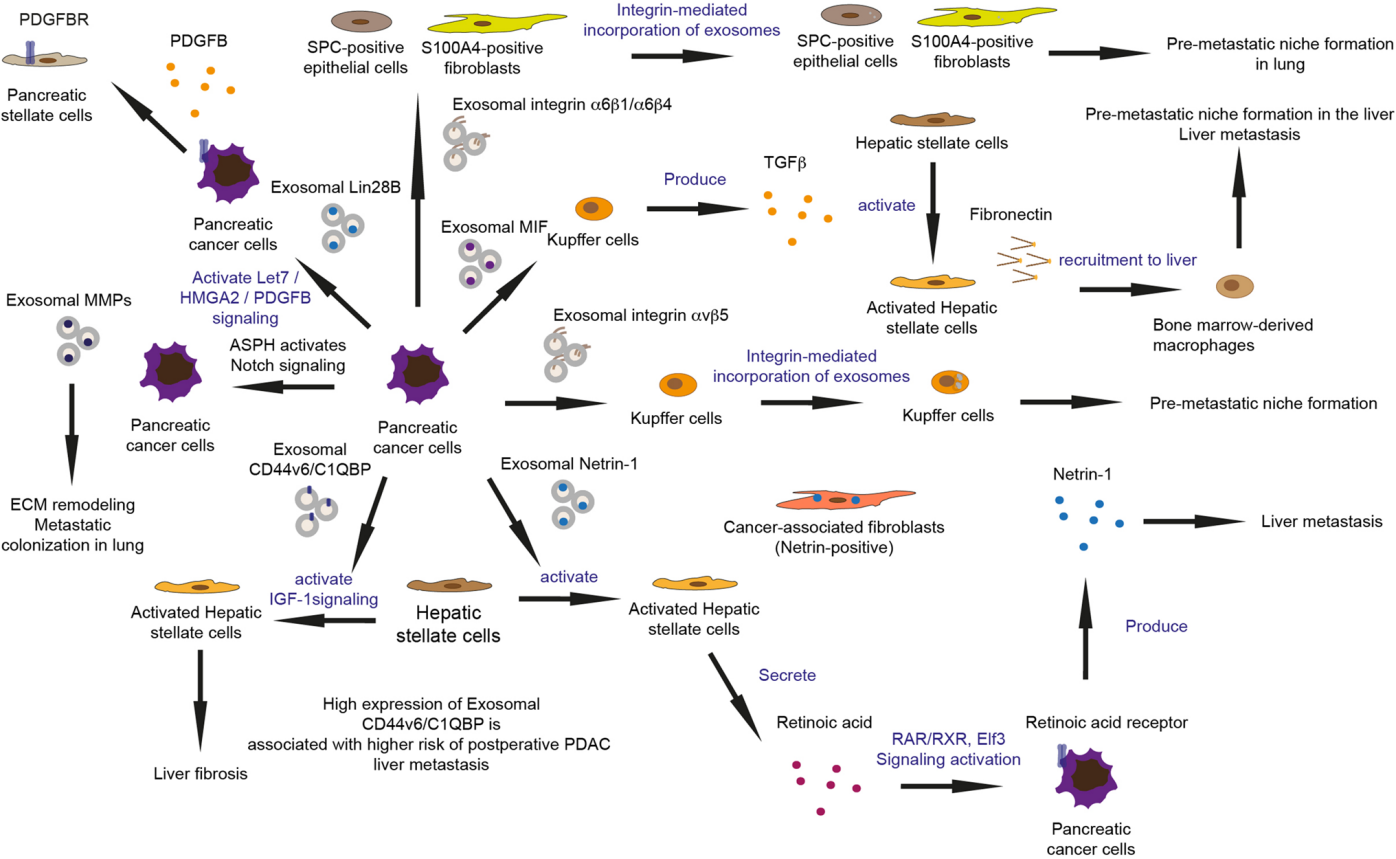
Pancreatic cancer-derived exosomes corporate with variety of stromal cells and promote liver and lung metastasis

There are several specific exosomes that can contribute to liver metastasis in pancreatic cancer via forming an inflammatory and immunosuppressive microenvironment in the liver, e.g., macrophage migration inhibitory factor (MIF) [49], Integrin ITG αvβ5 [50], Netrin-1 [28], Lin28B [51, 52] and CD44 [53, 54] (Fig. 1).

PDAC-derived exosomal MIF can be taken up by Kupffer cells in the liver, which then promotes TGF-β secretion, which may lead to the secretion of fibronectin by hepatic stellate cells (HSCs). Fibronectin subsequently makes a contribution to the recruitment of bone marrow-derived macrophages and neutrophils in the liver, thus forming the pre-metastatic niche [49]. The PDAC-derived exosomal ITG αvβ5 can also fuse with liver Kupffer cells, activating fibrotic pathways and creating a pre-metastatic niche conducive to cancer cell colonization (Table 2, Fig. 1) [50]. Netrin-1, transported via EVs from pancreatic cancer cells, initiates the activation of HSCs, culminating in liver metastasis. The process involves a complex signaling cascade, including retinoid and E74 like ETS transcription factor 3 (ELF3) pathways, which are crucial for the enhancement of metastatic cancer cell survival and proliferation within the liver [28]. PDAC-derived exosomal Lin28B enhances the recruitment of PSCs thereby promoting liver metastasis. The mechanism through which Lin28B in exosomes activates the Lin28B/let-7/HMGA2/PDGFB signaling pathway in recipient pancreatic cancer cells. The recipient pancreatic cancer cells produce platelet-derived growth factor β (PDGFB), and recruit PSCs via PDGF receptor. This cascade facilitates liver metastatic progression [51, 52]. CD44v6/C1QBP-loaded exosomes from pancreatic cancer cells activate IGF-1 signaling in HSCs supporting liver fibrosis, enhance tumor cell motility and activate host cells in the liver, promoting the establishment of a pre-metastatic niche conducive to liver metastasis. High expression of exosomal CD44v6/C1QBP is associated with higher risk of postoperative PDAC liver metastasis (Table 2, Fig. 1) [53, 54]. Taken together, pancreatic cancer-derived exosomes promote the formation of a pre-metastatic microenvironment through a series of complex signaling pathways, thereby enhancing the possibility of liver metastasis.

**Table 2:** Exosomes which contribute to organ-specific metastasis

Biomarkers	Donor	Recipient	Exosome function	Refs
Macrophage migration inhibitory factor (MIF)	Tumor cells	Kupffer cells (KCs) in liver	Liver metastasis	[49]
Netrin-1	Tumor cells	Hepatic stellate cells	Liver metastasis	[28]
Integrin αvβ5	Tumor cells	KC	Liver metastasis	[50]
CD44	Tumor cells	/	Liver metastasis	[53]
Lin28B	Tumor cells	Tumor cells/PSCs	Liver metastasis	[51, 52]
CD44v6/C1QBP	Tumor cells	/	Liver metastasis	[54]
Aspartate β-hydroxylase (ASPH)	Tumor cells	/	Lung metastasis	[55]
Integrin α6β4	Tumor cells	/	Lung metastasis	[56]
Integrin α6β1	Tumor cells	Lung fibroblasts	Lung metastasis	[50, 56]

### Lung metastasis

Lung metastasis in pancreatic cancer is considered less common than liver metastasis but still signifies advanced disease. The mechanisms underlying lung metastasis involve complex interactions between cancer cells and the pulmonary microenvironment. These may include alterations in the migration and invasion abilities of tumor cells, evasion of immune surveillance, and the formation of a pro-inflammatory microenvironment in the lung.

Exosomal aspartate β-hydroxylase (ASPH) is a pan-cancer biomarker, and it can be identified in biological fluids such as plasma or serum [57]. Exosomal ASPH supports metastatic establishment and expansion in lungs in murine models using orthotopic and tail vein injections of breast cancer cell lines [58]. In pancreatic cancer, ASPH enhances the secretion of exosomes from MIA-Paca2 cells, which carry components, like MMP2, that promote invasion/metastasis and immunosuppression via activating the ASPH-Notch signaling pathway, therefore leading to pulmonary metastasis in patient derived xenograft (PDX) murine models (Fig. 1) [55].

In distant metastasis of pancreatic cancer, the integrin family plays a crucial role by mediating interactions between tumor cells and the microenvironment of specific organs. Integrins constitute a class of transmembrane receptors that regulate cell adhesion to the ECM, impacting cell migration, survival, and proliferation. Integrins can recognize and bind to specific components of the extracellular matrix, such as fibronectin, collagen, and laminin, which are critical for the adhesion and localization of tumor cells in a new microenvironment. By promoting interactions between tumor cells and these matrix components, integrins facilitate the implantation and spread of tumor cells in organs like the liver or lung. In the process of distant metastasis in pancreatic cancer, different integrins play various roles. Exosomal integrins dictate the organotropic nature of metastasis by adhering to specific ECM components found in target tissues. This selective adhesion is crucial for the formation of a pre-metastatic niche which is a favorable microenvironment that supports the growth of metastatic tumor cells arriving at the site. Integrin αvβ5 in liver-tropic exosomes interacts with fibronectin, a major component of the liver ECM. As described before, this guides the exosomes to fuse with Kupffer cells, crucial for forming a pre-metastatic niche in the liver. However, Integrins α6β4 and α6β1 in lung-tropic exosomes interact with laminin, which is abundant in the lung ECM. This interaction facilitates the fusion of exosomes with lung-specific cells and helps in preparing the pre-metastatic niche in the lung (Table 2, Fig. 1) [50]. Integrin α6β4 in the exosomes is secreted by PDAC cells that have undergone PRKD1 loss, which leads to increased exosome secretion [56]. Exosomal Integrin α6β4 can promote lung metastasis; this effect was confirmed through exosome injection into non-obese mice with diabetes/severe combined immunodeficiency (NOD/SCID) xenograft mice, demonstrating the critical role of integrin α6β4 carried by exosomes in directing metastatic behavior specifically to the lung. Integrins α6β1 in tumor-derived exosomes also play a crucial role in directing pancreatic cancer metastasis to the lungs. Integrins α6β1 interacts with specific cells such as SPC-positive epithelial cells and S100A4-positive fibroblasts in the lung, facilitating organotropic metastasis through the activation of pro-inflammatory S100 genes, which are associated with the metastatic process (Fig. 1) [50]. This interaction prepares the pre-metastatic niche in the lungs, enhancing the metastatic potential of pancreatic cancer cells to this specific organ.

## The dual role of extracellular vesicles in drug resistance and therapeutic cargo delivery

**Fig. 2:**
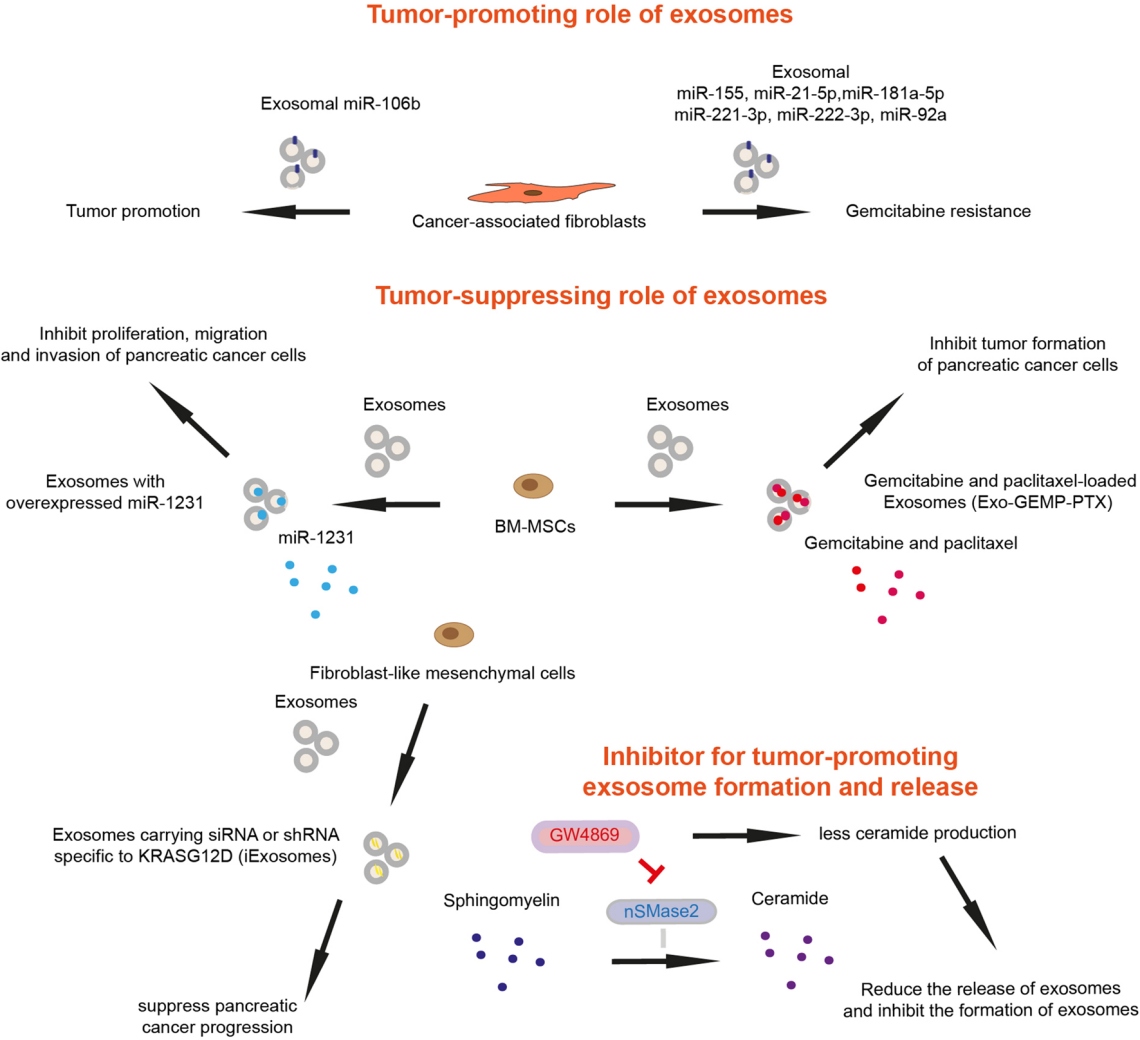
The dual role of exosomes in pancreatic cancer and an inhibitor for tumor-promoting exosome formation and release. The inhibition symbol is colored in red

There are some exosome-mediated cross-talks between pancreatic cancer cells and fibroblasts/CAFs [48]. Pancreatic cancer cells derived exosomes containing miR-155 can induce the conversion of normal fibroblasts to CAFs by altering gene expression, specifically by downregulating TP53INP1 expression and upregulating the expression of alpha-smooth muscle actin (α-SMA), fibroblast activation protein (FAP), and fibroblast growth factor 2 [36, 37]. CAFs secreting miR-155-containing exosomes can subsequently promote tumor progression, resulting in shorter survival of pancreatic patients (Fig. 2) [38]. The level of miR-155 in pancreatic cancer cells derived exosomes is also related to chemoresistance. Increased levels of miR-155 induced by gemcitabine can be transferred to other PDAC cells, offering protection from cell death caused by gemcitabine in both in vitro conditions and Nod/SCID mice [39–41]. There are several micro RNAs known to be associated with gemcitabine resistance, such as miR-21-5p, miR-181a-5p, miR-221-3p, miR-222-3p, and miR-92a [35]. The use of pharmacological approaches to block the secretion of exosomes from CAFs significantly reduces the survival rate of pancreatic cancer cells during gemcitabine treatment [24]. For example, GW4869 is one of the most commonly used drugs to reduce exosome release and inhibit exosome formation, it has the ability to block neutral sphingomyelinase 2 (nSMase2), a crucial regulatory enzyme that converts sphingomyelin into ceramide, which is required for the formation of exosomes (Fig. 2) [59]. CAFs can also secrete exosomes which can mediate tumor-promoting effects, and contribute to a worse prognosis in PDAC patients [24, 41]. The transport of miR-106b via exosomes originating from CAFs inhibit apoptosis by interacting with TP53INP1 in PDAC cells, although the precise process remains to be elucidated [13]. CAF-derived exosomes can also increase the chemotherapy resistance to gemcitabine treatment.

Interestingly, exosomes in pancreatic cancer microenvironment do not act only as tumor promoters, but also as tumor suppressors. Given that the recent advance in understanding biogenesis, secretion, and uptake of exosomes in target cells, engineered EVs have been considered as therapeutic tools in different cancers, including pancreatic cancer. In one study, exosomes were isolated from bone marrow-derived mesenchymal stem cells (BM-MSCs) and loaded with paclitaxel and gemcitabine monophosphate (named Exo-GEMP-PTX). Administration of Exo-GEMP-PTX inhibits xenograft tumor formation of pancreatic cancer cells and extends mouse survival (Fig. 2) [60]. Gemcitabine-loaded autologous exosomes (pancreatic cancer cell-derived exosomes) inhibited xenograft tumor formation of pancreatic cancer cells more effectively than gemcitabine administration [61]. Chemotherapeutical agent-loaded exosomes can be considered for treating pancreatic cancer patients. Since natural EVs are mostly retained in the liver or spleen, engineering strategies are important to increase targeting specificity and the chemotherapeutic efficacy [62]. Beside chemotherapeutic drug delivery, one study considered modification of micro RNA miR-1231 in exosomes as a targeting strategy in pancreatic cancer. It has been shown that low expression of miR-1231 in peripheral blood exosomes is associated with more advanced TNM stage of pancreatic cancer patients [63]. Administration of BM-MSC-derived exosomes overexpressing miR-1231 inhibits proliferation, migration, and invasion of pancreatic cancer cells (Fig. 2). Further, exosomes with overexpressed miR-1231 suppresses xenograft tumor growth of pancreatic cancer cells [63]. Mutations in *KRAS* gene are the most abundant (more than 90%) genetic alterations in pancreatic cancer patients [64]. Hence, targeting cells with KRAS mutations by small interfering RNA (siRNA), short hairpin RNA (shRNA), or KRAS mutation-specific small molecule inhibitors can be considered as tumor cell-specific targeting strategy with less off-target side effects. Exosomes derived from normal fibroblast-like mesenchymal cells carrying siRNA or shRNA specific to KrasG12D (named iExosomes) (Fig. 2) suppress pancreatic cancer progression and extend mouse survival in *Pdx1-Cre; lox-stop-lox-Kras*^*G12D/*+^*; lox-stop-lox-Trp53*^*R172H/*+^ (KPC) and *Ptf1a-Cre; lox-stop-lox-Kras*^*G12D/*+^*; Tgfbr2*^*lox/lox*^ genetically engineered pancreatic cancer mouse models [65]. For treating patients with metastatic pancreatic cancer with KRASG12D mutation, iExosomes have been entered into a clinical trial and a phase 1 study has been registered (NCT03608631) [66]. Thus, exosomes have dual role either in chemoresistance in pancreatic cancer, or chemotherapeutic cargo delivery. Engineered exosomes as carriers for chemotherapeutic drugs and targeted RNA demonstrate potential for treating pancreatic cancer and its metastasis, including strategies targeting *KRAS* gene mutations, offering new directions for therapy.

## Conclusion

Pancreatic cancer/CAFs-derived exosomes emerge as key players in the pancreatic cancer microenvironment, mediating complex intercellular communications that significantly influence cancer proliferation, invasion, progression, organ-specific metastasis, and resistance to therapy. Targeting exosomal pathways presents a promising therapeutic avenue, offering hope for more effective treatments. Exosomes are also considered promising therapeutic cargo delivery system targeting not only primary pancreatic cancer, but also metastasis. Future research should prioritize unraveling the intricate mechanisms of exosome function and interaction, aiming to harness their full potential in the battle against pancreatic cancer. This exploration could unlock groundbreaking advancements in diagnosis, treatment, and ultimately, patient survival rates.

## Data Availability

No datasets were generated or analysed during the current study.
